# Measuring health-related quality of life for child maltreatment: a systematic literature review

**DOI:** 10.1186/1477-7525-5-42

**Published:** 2007-07-16

**Authors:** Lisa A Prosser, Phaedra S Corso

**Affiliations:** 1 Center for Child Health Care Studies, Harvard Medical School and Harvard Pilgrim Health Care, Department of Ambulatory Care and Prevention, Boston, MA, USA; 2 College of Public Health, University of Georgia, Athens, GA, USA

## Abstract

**Background:**

Child maltreatment causes substantial morbidity and mortality in the U.S. Morbidity associated with child maltreatment can reduce health-related quality of life. Accurately measuring the reduction in quality of life associated with child maltreatment is essential to the economic evaluation of educational programs and interventions to reduce the incidence of child maltreatment. The objective of this study was to review the literature for existing approaches and instruments for measuring quality-of-life for child maltreatment outcomes.

**Methods:**

We reviewed the current literature to identify current approaches to valuing child maltreatment outcomes for economic evaluations. We also reviewed available preference-based generic QOL instruments (EQ-5D, HUI, QWB, SF-6D) for appropriateness in measuring change in quality of life due to child maltreatment.

**Results:**

We did not identify any studies that directly evaluated quality-of-life in maltreated children. We identified 4 studies that evaluated quality of life for adult survivors of child maltreatment and 8 studies that measured quality-of-life for pediatric injury not related to child maltreatment. No study reported quality-of-life values for children younger than age 3.

Currently available preference-based QOL instruments (EQ-5D, HUI, QWB, SF-6D) have been developed primarily for adults with the exception of the Health Utilities Index. These instruments do not include many of the domains identified as being important in capturing changes in quality of life for child maltreatment, such as potential for growth and development or psychological sequelae specific to maltreatment.

**Conclusion:**

Recommendations for valuing preference-based quality-of-life for child maltreatment will vary by developmental level and type of maltreatment. In the short-term, available multi-attribute utility instruments should be considered in the context of the type of child maltreatment being measured. However, if relevant domains are not included in existing instruments or if valuing health for children less than 6 years of age, direct valuation with a proxy respondent is recommended. The choice of a proxy respondent is not clear in the case of child maltreatment since the parent may not be a suitable proxy. Adult survivors should be considered as appropriate proxies. Longer-term research should focus on identifying the key domains for measuring child health and the development of preference-based quality-of-life instruments that are appropriate for valuing child maltreatment outcomes.

## Background

Child maltreatment, typically defined as physical abuse, sexual abuse, emotional abuse, physical neglect, and/or emotional neglect of children ages 0–17 years by their parents or caretakers, causes substantial morbidity and mortality in the United States. The most recent report estimates that approximately 906,000 children were maltreated in the US in 2003, and an estimated 1,500 children were confirmed to have died from their injuries[1]. Estimates of the lifetime economic impact of child maltreatment, as measured by direct medical costs and productivity losses, have been estimated as $93 billion[2]. Although large, these estimates likely represent an underestimate of the true burden of child maltreatment due to the lack of accurate incidence reporting and the inability to assess costs for indirect effects of child maltreatment on temporary or permanent physical and cognitive disabilities, and sustained losses in future education and occupational attainment[3]. Furthermore, there is evidence that child maltreatment can cause an increase in risky behaviors later in life such as smoking, alcoholism, drug use, eating disorders, obesity, depression, suicide, sexual promiscuity and specific chronic diseases [4-9]. Maltreatment that occurs during infancy or early childhood such as shaken-baby syndrome (SDS) can similarly lead to life-long physical, mental and cognitive impairments because the brain has been damaged[10].

This broad range of child maltreatment's impact on health suggests that it may also have a substantial impact on its victims' life expectancy and long-term health-related quality of life (HRQL). HRQL can be measured using either health status measures or preference-based measures, with each category providing different information. Health status measures summarize the presence, absence, severity, frequency, and/or duration of specific symptoms, impairment, or disabilities. Examples of health status measures used in children include the Pediatric Quality of Life Inventory (Peds-QL), Child Health and Illness Profile (CHIP), and the Child Health Questionnaire (CHQ) [11,12]. These measures provide information on several domains of HRQL. In contrast, preference-based measures provide a summary value for a respondent's valuation of the quality of life of a particular health state, incorporating all positive and negative aspects of a health state into a single number. A commonly used approach for valuing preferences in health is utility. A utility value is typically scaled between 1.0, representing perfect health, and 0.0, representing a health state judged equivalent to being dead (although health states worse than dead can be scaled less than zero). Economic evaluations require the use of a preference-based measure of health states to correctly value changes in health [13-15].

Cost-effectiveness analysis, a type of economic evaluation, is one of the most widely used tools available to policy makers to measure the relative value of health care interventions. Cost-effectiveness analyses can be used by health insurers as well as federal and state policy makers as important guides to the allocation of resources. They provide the cost per health benefit gained from any single health intervention and also help to demonstrate which of two competing health care interventions is a better value[16]. Cost-utility analysis, a subset of cost-effectiveness analysis, uses a generic measure of benefit, the quality-adjusted life year (QALY), to measure the amount of health benefit associated with an intervention. The quality of a cost-utility analysis depends in large part on the quality of the inputs into the analysis as well as on the validity of the structure of the model. These inputs typically fall into three categories: probabilities, costs, and quality adjustments for health outcomes (to calculate QALYs gained). Therefore, the validity of a cost-effectiveness analysis will depend upon the appropriate valuation of health benefits.

In the context of child maltreatment, economic evaluation can be used to answer key questions, such as, are home visitation programs cost-effective relative to alternative interventions designed to reduce child maltreatment? Do these programs provide a positive return on investment, and from whose perspective? Are programs, that are provided in different settings, with different practitioners, to different target populations, delivered in a cost-efficient manner?

Scant research has focused on measuring HRQL for maltreated children; yet, to evaluate the total effectiveness of intervention/prevention programs for child maltreatment, accurate measures of the effect of child maltreatment on HRQL must be utilized that capture changes in all relevant domains of health. In addition, these measures need to appropriately capture the long-term impacts of child maltreatment that can occur in adulthood.

This study reviews the available literature on HRQL associated with child maltreatment using either preference-based, health status, or other approaches. There are two general approaches to measuring preference-based quality of life for economic evaluations: direct assessment or multi-attribute utility instruments. We will consider advantages and disadvantages of each of these approaches for measuring HRQL for child maltreatment. We will also describe challenges specific to eliciting utilities from children such as cognitive challenges of the task, using parent as proxies, defining health differently than from adults, using community- level weights (or tariffs) that have been elicited from adult populations and applying them to child defined health states. Recommendations for next steps and future research are detailed.

## Methods

### Literature search and study selection

Literature searches were conducted to identify (1) studies that measured HRQL for child maltreatment outcomes and economic evaluations of child maltreatment prevention or intervention programs, and (2) existing multi-attribute and health status instruments for children and adults. Three databases were utilized: PubMed, Econlit, and PsychInfo. Search terms and search strategies are listed in Appendix 1 [see Additional file 1].

### Study inclusion/exclusion

The search for studies that measured HRQL for child maltreatment was limited to literature from English speaking countries and published between the years 1976–2006. At the first stage, article titles were reviewed and any articles that had HRQL, preferences and/or economic evaluation were included. In the next stage, abstracts were read to confirm that studies were relevant. A complete reading of the remaining articles was done to verify that all the papers contained the appropriate information. Articles were excluded at this stage, if subjects were greater than 17 years of age and/or did not meet the criteria listed above.

A data abstraction form (Appendix 2) [see Additional file 2] was specifically created to summarize all significant information from articles and to collect data in an organized fashion that would be simple to analyze. The abstraction form contained information on descriptive elements of the study, including: title of the article, authors' names, name of the journal and year it was published, country of origin, time frame, type of maltreatment, type of study (i.e. health status, preference-based, descriptive), how preferences were measured, and HRQL instruments utilized. The form assisted in the collection of specific methodologic information and categorized data elements into the following items: source of preferences (i.e. patient, parent, expert etc.), age of respondents, technique used in valuing preferences such as direct (i.e. rating scale, standard gamble, time trade off) and indirect (i.e. Health Utilities Index, Quality of Well-Being scale, EuroQol-5D) assessment of preferences, identification of the rater of health states in the study (i.e. patient, parent, and expert), age of rater, if the raters experienced health states and if so, how long were the health states and what were the health states, and age of the child whose HRQL was assessed.

The detailed information collected in the abstraction form allowed us to assess methods for calculating QALYs, observe which techniques and instruments were used to measure preferences as well as whose quality of life was assessed, and to identify from whom values were obtained.

The literature was then reviewed to identify generic quality of life instruments that could be applied to child maltreatment. The instruments reviewed were the Euroqol 5 dimension scale (EQ-5D)[17], the Health Utilities Index (HUI)[18], the Quality of Well Being scale (QWB)[19], and the Short Form 36 (SF-36)[20]. Pediatric HRQL instruments such as Pediatric Quality of Life Inventory (Peds-QL)[21], Functional Status II (FS II)[22], Health Status Questionnaire (HSQ)[23], Child Health Questionnaire (CHQ)[24], and Child Health and Illness Profile (CHIP-CE)[12] were also investigated to ascertain which domains were covered and if they were applicable to child maltreatment. The following information was collected: a description of the instrument, including domains and attribute levels of health, target population and target health outcome, a description of reliability and validity testing, and scoring system, if available.

## Results

### Literature review

The original search identified 1960 candidate articles from PubMed (91%), PsychInfo (8%), and EconLit (1%). At the first stage, 1573 articles were eliminated because they did not meet the inclusion criteria and/or were duplicate papers. At abstract review, another 364 were eliminated because they did not include measures of HRQL related to child maltreatment. 23 papers were retrieved for full review and an additional 7 were eliminated as they did not include measures of HRQL related to child maltreatment. Due to the small number of papers, we also retained studies that measured HRQL for outcomes that might be related to child maltreatment (e.g., injury) and for adult survivors of child maltreatment.

All of the studies were published since 1990 and all except one were conducted in the United States. The CHQ and SF-36 were each used in 3 studies; "monetized QALYs" in 2 studies; the Vineland scale and Peds-QL were each used in one study; and other approaches used in 2 studies. Age of the subject's health being valued was 3 years or greater in all studies. Respondents had experienced the health state in half of the studies.

### Types of studies

We did not identify any papers that directly measured HRQL for child maltreatment outcomes using standard gamble, time-tradeoff or rating scale methodologies. We identified 4 papers that indirectly measured HRQL for adult survivors of child maltreatment[3,25-27], 8 papers that measured HRQL for pediatric injury not specifically related to child maltreatment [25,28-33], and 4 papers that reported costs for hospitalizations or investigations related to suspected abuse [34-37]. Key data elements abstracted from these 12 papers are listed in Appendix 3 [see Additional file 3].

Of the studies that evaluated HRQL for adult survivors of child maltreatment, 3 used the SF-36 [25-27] and one developed their own survey that included questions on health status, leisure-time physical activity, depressed mood and suicide attempts from other validated questionnaires including the Conflicts Tactics Scale, the National Health Interview Survey, the Behavioral Risk Factor Surveillance Survey, and the National Health and Nutrition Examination Survey [3,38]. No study used a validated preference-based QOL instrument such as the EQ-5D, HUI, or QWB. While it is possible to derive utility scores from the SF-6D (a subset of questions from the SF-36) using an algorithm developed by Brazier et al.[39], this approach was not utilized in any of the published studies.

Of the 8 studies that measured HRQL for pediatric injuries not specifically related to maltreatment, 3 used an approach consistent with preference-based measurement. One used the QWB and 2 used "monetized QALYs". In the 2 studies that used monetized QALYs, injuries were scored using the Functional Capacity Index and translated to a loss in QALYs using a combination of utility scores and rating scale estimates from 5 sources (HUI-1, QWB, jury awards, expert opinion, and student ratings). The number of QALYs lost was then multiplied by the willingness-to-pay for an additional QALY derived from estimates of the value for a statistical life[28,40,41]. One study used the QWB to report health status scores but did not use the QWB to calculate utility weights [25]. Three studies used the CHQ to measure HRQL for injury (all types) and traumatic brain injury [30,31,33]. One study used the Peds-QL to value quality-of-life for traumatic brain injury [32]. One was a review of pediatric quality-of-life instruments that could be used to evaluate pediatric injury [29].

The 4 cost studies reported costs of hospitalizations for burns [35], pediatric intensive care admissions for child maltreatment [36], poison [34], and investigation of suspected abuse [37]. We also identified a benefit-cost analysis of a nurse home visitation program to improve child health outcomes, but this was conducted from the government perspective and included direct government costs only[42]. None of these studies included measures of HRQL and are not considered further in this review. Health domains relevant to child maltreatment were identified through the literature review and expert opinion (Table 1).

**Table 1:** Health domains relevant to quality-of-life for child maltreatment.

**Category**	**Domains**	**Specific descriptions**
Growth and development	Developmental progress/opportunity for normal development	Gross motor delays Fine motor delays Speech and language delays Learning disabilities
	Communication and social interaction through play	Communication Learning Social skills
Functioning	Feeding	Feeding issues Malnutrition
	Dressing	
	Bathing	
	Toilet constancy	Continence
	Mobility	
	Attending school	Ability to function in school Truancy
	Interacting with peers	
Physical Health	Routine medical care	Immunization delay Lack of well child care Dental caries
	Physical Function	Permanent or semipermanent Limb deformity, contractures Brain injury (seizure disorders, visual impairment)
Emotional Well-being/Mental Health	Anxiety	Post-traumatic stress disorder
	Depression	
	Anger	
	Risky behavior	Promiscuity, teen pregnancy, sexually transmitted infections, juvenile delinquency (incarceration), suicide, smoking, alcohol abuse, drug abuse
	Other psychiatric disorders	Obesity, eating disorders, body dysmorphic disorders, GI disorders
Placement and family stability	Function within a family	Attachment disorders
	Impact on parents/caregivers	
	Foster care, adoption	

### Generic qualify of life instruments

Five pediatric quality-of-life instruments (non-preference-based) and six adult preference-based quality-of-life instruments were identified. Characteristics of pediatric quality-of-life instruments are detailed in Table 2. Domains of generic pediatric and adult quality-of-life instruments are listed in Table 3.

**Table 2:** Characteristics of pediatric quality-of-life instruments.

Instrument	**Domains**	**Number of Items**	**Time to complete (approximate)**	**Ages Intended For**	**Respondent**	**Year Developed**
Peds-OL	3 Domains -Physical Functioning -Psychological Functioning -Social Functioning	15 items	5–10 minutes	2–7 years 8–12 years 13–16 years	Parent/proxy Child	1998
FS II	8 Domains -Communication -Mobility -Mood -Energy -Play -Sleep -Eating -Toilet patterns	43 items (long version) 14 items (short version)	30 minutes	0–16 years	Parent	1978
CHSQ	5 Domains -Physical Health -Mental Health -Social Relations -General Health -Satisfaction with development	34 items	10–15 minutes	2 year +	Pediatrician form Parental form	1994
CHQ	14 Domains -Physical Functioning -Social Functioning -Bodily/Discomfort -General Behavior -Mental Health -General Health Perception -Self Esteem -Parental Impact (time & emotional) -Family Functioning (actives & cohesion)		20 minutes	5–13 years 10–18 years	Parent/Proxy (CHQ-PF-98; PF-50; PF-28) Children (CHQ-CF-87) No parallel versions	1990 1994–1995
CHIP	7 Domains -20 sub domains -Satisfaction -Comfort -Resilience -Risk Avoidance -Achievement -Disorders -Home Safety & Health	107 items 46 specific to disease or injury	30 minutes	6–11 years (CE) 11–18 years (AE)	Parent/Proxy Child	1993

**Table 3:** Domains included in multi-attribute utility and pediatric quality-of-life instruments.

	**Generic QOL instruments with utility weights**						**Pediatric QOL instruments**					**Relevant to maltreatment**
**Domains Covered**	**EQ-5D**	**HUI 2**	**HUI 3**	**QWB**	**SF-36**	**SF-6D**	**PedsQL**	**FS II**	**CHSQ**	**CHQ**	**CHIP**	**Domains specific to child maltreatment**

Physical Functioning/Physical Health	X	X	X	X	X	X	X	X	X	X	X	X
Mobility	-	-	-	-	-	-	-	X	X		X	X
Pain/Discomfort	X	-	X	-	X	X	X	-	-	X	X	X
Self Care	-	X	-	-	-	-	X	-	-	-	-	-
Sleep/Rest	-	-	-	-	-	-	X	X	-	-	-	-
Energy/Vitality	-	-	-	-	-	X	X	X	-	-	-	-
Toilet patterns	-	-	-	-	-	-	-	X	-	-	-	X
Cognitive Functioning	-	X	X	-	-	-	X	-	-	-	-	-
Symptoms (impairments)	-	X	X	X	-	-	-	-	X	-	-	-
Sensory function (loss)	-	X	X	-	-	-			X	-	-	X
Respiratory Function	-	-	-	-	-	-	-	-	X	-	-	X
Renal Function	-	-	-	-	-	-	-	-	X	-	-	-
Perceptions in Health	-	-	-	-	X	-	-	-	-	-	X	X
Social Functioning	-	X	-	X	X	X	X	X	-	X	X	X
Other: Recreation/Past Times/Activity	X	-	-	-	X	-	X	X	-	X	-	X
Work/School	-	-	-	-	X	X	X	X	-	X	X	X
Home Management	-	-	-	-	X	-	-	X	-	-	-	-
Eating	-	-	-	-	-	-	-	X	X	-	-	X
Level of Independence	X	-	-	-	-	-	-	-	-	-	-	-
Relationship	-	-	-	X	-	-	-	-	-	X	X	X
Interactions	-	-	-	-	-	-	X	-	-	X	X	X
Emotional Functioning/Metal Health/Well Being	X	X	X	X	X	X	X	-	-	X	X	X
Other: Self-Image/Self Esteem	-	-	-	-	-	-	X	-	-	X	-	X
Behavior	-	-	-	-	-	-	-	-	-	X	X	X
Distress/Health-Related Distress	-	-	-	-	-	-	X	-	-	-	X	-
Life Satisfaction/Overall QoL	X	-	-	-	-	-	-	-	-	-	X	-
Communication	-	X	X	-	-	-	X	X	X	X	-	-
Parental Impact	-	-	-	-	-	-	-	-	-	X	X	-
Family Activities	-	-	-	-	-	-	-	-	-	X	X	-
Family Function/Dynamics	-	-	-	-	-	-	-	-	-	X	X	X
Overall Growth & Development	-	-	-	-	-	-	-	-	X	X	-	X
Achievement-Academically & Socially	**-**	**-**	**-**	**-**	**-**	**-**	X	**-**	**-**	**-**	X	X
Home Safety & Health	**-**	**-**	**-**	**-**	**-**	**-**	-	**-**	**-**	**-**	X	X

## Discussion

This review underscores the scant attention that has been paid to measuring HRQL associated with child maltreatment outcomes. One reason for the paucity of data is likely due to challenges in accurately measuring preference-based quality-of-life in children including limits to the approaches typically used for valuing adult quality of life. Other potential reasons for the scarcity of data on this topic could include the difficulty in obtaining IRB approval for such a study, the morbid nature of the area and researchers' willingness to study this area. Preference-based approaches for measuring the loss in health-related quality of life due to child maltreatment should be considered in the context of the general challenges of valuing preferences for children's health and additional challenges specific to child maltreatment.

### Challenges to valuing loss in HRQL for child maltreatment

A key challenge to valuing children's health for economic evaluations as compared to adults is the necessity of using a proxy respondent. Most children will need proxy respondents to complete value elicitation exercises. Prior studies have demonstrated that standard gamble and time trade-off surveys can only be reliably completed by children with at least a 6^th ^grade reading level [43] indicating that proxies will be required for most children 12 or younger. Adolescents may be able to value their own health, while school-age, preschool, and infants will require proxy respondents. Adolescents can complete such exercises but may demonstrate inconsistencies with using these valuation methods. When adolescents' values were compared to adult values for the same health states, adolescent values were significantly different than values elicited from parents or other adults[44,45]. An additional challenge for child maltreatment is that the parent/caregiver may be the abuser and while the parent may be the natural proxy for valuing other types of children's health, the parent/caregiver will often not be the appropriate proxy. In the case of child maltreatment where the parent may be the abuser or may also be affected by abuse, an alternate proxy may be preferable. The optimal respondent may be adult survivors of maltreatment or adolescents affected by maltreatment. Using adult survivors may also be related to other biases such as recall bias or adjustment bias. If an adult survivor has overcome early child abuse or has many resulting health problems could potentially affect how one recalls and values the maltreatment experience.

The age of a child during the health state also poses methodological and practical challenges to valuing child health. For the same illness, the valuation of a health state may vary depending on the age of the affected person holding all other aspects of health constant. For example, maltreatment domains could vary between infants and adolescents as the most prevalent type of maltreatment tends to vary by age[46]. The long-term impacts of child maltreatment in adulthood should also be measured; several studies have demonstrated an impact on quality of life in adults[3,38,47]. This issue must be considered in the design or use of multi-attribute instruments.

None of the existing preference-based or pediatric QOL instruments address QOL for children under 2, and this age group poses significant challenges for measuring QOL. Some have advocated an approach that includes spillover effects on other family members [48], but such an approach may be inconsistent with valuing child maltreatment since the parent/caregiver may be the abuser. Thus, methods for valuing child maltreatment outcomes may depart from the development of general methods for valuing children's health.

There are additional challenges specific to measuring quality-of-life for child maltreatment (violence) that include difficulty in defining the severity of violence in a consistent way, changing pattern of maltreatment over time, and the potential exclusion of parent/caregiver as a candidate for proxy respondent. The increased prevalence of child maltreatment among children with disabilities [49] also presents additional problems in measuring QOL in already-disabled children.

### Strengths and limitations of existing approaches to valuing health

Preferences for child maltreatment health states can be derived using one of the available multi-attribute utility instruments (EQ-5D, HUI, QWB) [17-19]. The most appropriate respondents using this approach would be adolescents or adult survivors valuing experienced health states. The EQ-5D has the most representative sample but does not include some key domains for valuing child health. The HUI and QWB include additional domains but utility weights are based on small and non-representative samples.

A number of instruments have been developed to measure quality of life in children but are not preference-based. One research option would be to develop utility weights for one or more of these instruments similar to the approach used for the SF-6D. The advantage of deriving utility weights for an existing instrument is that many of these instruments already include domains specific to child health and child maltreatment and have been validated for children. The Peds-QL also offers age-specific versions of its instrument for ages 2–7, 8–12, 13–16. Disadvantages of this approach are the lack of instruments for valuing infant health and that some domains specific to child maltreatment may be missing.

The direct valuation approach tends to be more resource-intensive than the use of a multi-attribute utility instrument but may produce more accurate measurements of quality of life associated with child maltreatment since the surveys can be designed to include all domains relevant to child maltreatment. Utilities could be elicited from adolescents or adult survivors valuing experienced health states or from a community sample valuing hypothetical health state descriptions of child maltreatment. Either "patient" or "community"-perspective utility weights can be elicited via direct valuation. Utilities for child maltreatment states of health can be directly assessed using either standard-gamble or time-tradeoff questions[13].

## Conclusion

Neither currently available preference-based HRQL instruments nor direct valuation approaches provide ready solutions for measuring preference-based quality of life in child maltreatment. Currently available preference-based HRQL instruments were developed primarily for chronic conditions in adults and may not fully capture loss in quality of life associated with domains of health relevant to child maltreatment or complex health states that include both temporary and chronic health events. Nevertheless, these instruments represent a good short-term solution. Direct valuation approaches present a number of methodologic and practical challenges when applied to pediatric populations but can be designed to better capture loss in quality of life compared to existing HRQL instruments.

For both approaches, the challenges of eliciting values will differ according to age groups: infant, preschool, school-age, and adolescent. In addition, direct valuation can be more expensive and time-consuming. It can also be more difficult logistically to obtain values from a representative national sample when using direct valuation.

This review did not originally focus on measuring quality of life for adult survivors of maltreatment but articles identified in the literature search were reviewed given the scarcity of literature on quality of life and child maltreatment at the time of maltreatment. Nor did we consider how psychological abuse in childhood could manifest as physical outcomes in adults. We also did not specifically include the effects of witnessing domestic violence, although consequences of witnessing domestic violence on physical and psychological outcomes could also be substantial.

In the short term, preference-based HRQL for child maltreatment from the community perspective can be most easily measured using the EQ-5D (or another preference-based HRQL instruments) with either adult survivors and/or adolescents who have experienced maltreatment. The sample used to derive utility weights for the EQ-5D is the most representative sample of the available preference-based QOL instruments for US policy decisions[50]. In addition, the EQ-5D covers at least 2 of the domains that are likely to be directly relevant to child maltreatment: physical functioning and emotional well-being. However, this approach will not be able to provide any quality-of-life effects specific to children (e.g., growth and development) and does not provide values for "experienced" utility.

If the objective is to measure experienced utilities (i.e., the "patient perspective"), this could be achieved using direct valuation measures with adult survivors or adolescents rating their own childhood experience. While adolescents who have experienced child maltreatment may be the most suitable proxy since they have direct experience with the health state and recall bias will be less than for older adult survivors since less time has passed since the experience, adolescents' responses may be biased as described above. In addition, respondent burden must be considered. If it is too emotionally burdensome for adolescents to answer these types of questions, other respondents must be considered.

A third alternative is to have a community sample rate hypothetical descriptions of child maltreatment. This would provide community weights that might better reflect quality of life associated with child maltreatment but will be more resource-intensive compared to using an existing preference-based HRQL instrument.

Longer-term research should focus on identifying the key domains for measuring child health. Identifying the domains that are most relevant to child maltreatment will allow for a more comprehensive evaluation of existing preference-based and pediatric HRQL instruments. Consideration should be given to how the different types of child maltreatment affect different domains of quality of life.

The conclusion of this review is that the existing pediatric HRQL instruments cover a more comprehensive set of domains relevant to children's health and specifically child maltreatment than the preference-based generic HRQL instruments. If this finding is confirmed, then the recommended approach would be to either (1) develop utility weights for one of the pediatric quality of life instruments or (2) design a new preference-based HRQL instrument to measure preferences for child health/maltreatment. The Peds-QL covers relevant domains and offers age-specific versions and could be a good candidate for the first approach. Any approach needs to consider the special characteristics of valuing child health. If children are to complete surveys themselves, the use of age-appropriate visual aids that improve reliability and validity of responses will be essential[51]. Long-term effects of child maltreatment should also be considered. Although maltreatment may occur during childhood, many of the negative consequences will affect a maltreated individual throughout the lifecourse, and this should be considered when developing appropriate measures. If an instrument were developed specifically for measuring effects of child maltreatment, then the optimal approach may be to develop an instrument that can measure HRQL related to child maltreatment for all ages, child to adult. Additional research is needed to improve methods for measuring preference-based quality of life in child maltreatment.

## Competing interests

The author(s) declare that they have no competing interests.

## Authors' contributions

LP led the design and literature search, designed the abstraction forms, supervised data abstraction, performed the analysis, and drafted the manuscript. PC conceived of the study, participated in its design and interpretation of results, and participated in drafting the manuscript. All authors read and approved the final manuscript.

## Supplementary Material

Additional file 1 Appendix 1: Search terms for child maltreatment literature review Click here for file

Additional file 2 Appendix 2: Child Maltreatment – Data Extraction Form Click here for file

Additional file 3 Appendix 3: Data abstracted from literature review Click here for file
