# Supplementary material for: Measuring health-related quality of life for child maltreatment: a systematic literature review
Source: Health Qual Life Outcomes. 2007 Jul 16;5:42. doi: 10.1186/1477-7525-5-42 (PMC1951964; doi:10.1186/1477-7525-5-42)
Supplement: Additional file 1 — Appendix 1: Search terms for child maltreatment literature review [file 1477-7525-5-42-S1.doc]

Appendix 1: Search terms for child maltreatment literature review

Condition:

1. Child abuse
2. Child maltreatment, including

- Sexual-rape, molestation, assault
- Psychological
- Physical
- Medical neglect

1. Pediatric injury

Type of study:

1. Health preferences, including
   - Utility
   - Willingness to pay
   - Rating scale
   - Time trade off
   - Standard gamble
   - DALY
2. Quality of life
   - SF-36
   - SF-6D
   - Peds-QL
   - CHIP-AE
3. Economic evaluation, including

- Cost-effectiveness
- Cost-benefit
- Cost-utility
