# Supplementary material for: Measuring health-related quality of life for child maltreatment: a systematic literature review
Source: Health Qual Life Outcomes. 2007 Jul 16;5:42. doi: 10.1186/1477-7525-5-42 (PMC1951964; doi:10.1186/1477-7525-5-42)
Supplement: Additional file 2 — Appendix 2: Child Maltreatment – Data Extraction Form [file 1477-7525-5-42-S2.pdf]

CHILD MALTREATMENT – DATA EXTRACTION FORM

Title (abbrev) ____ First Author ________________ Reviewer _____

STUDY INFORMATION

Country of Origin
- ❑ USA ❑ Canada ❑ United Kingdom
- ❑ Australia ❑ New Zealand ❑ Other ____________________

Time Frame ❑ Retrospective ❑ Prospective

Type of Maltreatment
- ❑ Physical ❑ Neglect ❑ Medical Neglect
- ❑ Sexual ❑ Psychological ❑ Other______________________

Type of Study:
- ❑ Preference (e.g.: utility, WTP) ❑ Quality of Life (pedi or Generic QoL Instrument) ❑ Descriptive (provides info on affected health attributes)
- ❑ Economic Evaluation ❑ Other____________________

Preference measured in
- ❑ QALY ❑ DALY ❑ Other ____________________
- ❑ WTP

QoL Instrument
- ❑ SF-36 ❑ Peds-QL ❑ Other___________________
- ❑ SF-6D ❑ CHIP-AE

SOURCE OF PREFERENCES

Preferences of:
- ❑ Patient ❑ Proxy ❑ Community ❑ Expert
- ❑ Parent ❑ Caregiver ❑ Other_______________

Age of source: Mean:________ Median: ___________ Range: _________________

TECHIQUE USED IN VALUING PREFERENCES

Direct assessment of preferences

❑ Rating Scale: ❑ Categories ❑ Numbers ❑ Line

❑ Standard gamble: Chronic / Temporary (circle one) health state _______________________________
Worst health state: ❑ Death ❑ Other _______________

❑ Time tradeoff: Time in current health state (denominator): ___________________________________

❑ Other method: Relevant details ____

Indirect assessment of preference

Multi-attribute util. ❑ HUI ( ) ❑ QWB ❑ EQ-5D ❑Other:___________

❑ Disease Specific______________________________

RATER OF HEALTH STATES IN STUDY (for generic and disease specific instruments)

Ratings of: ❑ Patient ❑ Proxy ❑ Community ❑ Expert ❑ Other___________

Age of rater: Mean: _________ Median: ___________ Range: __________________________

Did rater experience health states in study? ❑ Yes ❑ No

How long were the health states? ❑ Temporary ❑ Chronic ❑ Pathway

Health States Valued _____________________

CHILD INFORMATION

Whose QoL was assessed? ❑ Child ❑ Parent ❑ Both ❑ Other _________

Age of children (at start): Mean: _________ Median: ___________ Range: __________________________

OTHER INFORMATION

____

____

Pages of interest (optional entry): ___________________________________
